# Supplementary material for: Association of the CFTR gene with asthma and airway mucus hypersecretion
Source: PLoS One. 2021 Jun 4;16(6):e0251881. doi: 10.1371/journal.pone.0251881 (PMC8177500; doi:10.1371/journal.pone.0251881)
Supplement: S1 Appendix — (ZIP) [file pone.0251881.s001.zip › English Expectoration Questionnaire.docx]

**DATA COLLECTION SHEET**

**4. MUCOUS SECRETION DATA (select the answer that best defines your situation)**

**4.1 How often do you expectorate? (last 3 months)**

1. I expectorate every day

2. I only expectorate some days

3. I only expectorate with colds

4. I don't expectorate _____

**4.2 How much do you expectorate? (average last three months)**

1. One or 2 times a day

2. From 3 to 6 times a day

3. From 7 to 14 times per day

4. More than 15 times a day

5. More than 30 times a day _____

**4.3 If you expectorate, how many years have you been expectorating? _____**

**4.4 How would you define your expectoration**

1. I easily expectorate liquid mucus

2. I have very sticky mucus that is difficult to remove _____
