# Supplementary material for: Association of the CFTR gene with asthma and airway mucus hypersecretion
Source: PLoS One. 2021 Jun 4;16(6):e0251881. doi: 10.1371/journal.pone.0251881 (PMC8177500; doi:10.1371/journal.pone.0251881)
Supplement: S1 Appendix — (ZIP) [file pone.0251881.s001.zip › Spanish expectoration questionnaire.docx]

***HOJA DE RECOGIDA DE DATOS***

**4. DATOS SECRECIÓN MUCOSA (selecciona la respuesta que mejor defina tu situación)**

**4.1 ¿Con que frecuencia expectora? (últimos 3 meses)**

1. Expectoro cada día
2. Algunos días expectoro pero otros no
3. Solo expectoro con los resfriados
4. No expectoro _____

**4.2 ¿Qué cantidad de expectoración? (promedio últimos 3 meses)**

1. Uno o dos esputos al día

2. De tres a 6 esputos al día

3. De 7 a 14 esputos al día

4. Más de 15 esputos al día

5. Más de 30 esputos al día

_____

**4.3 Si expectora, cuántos años hace que expectora _____**

**4.4 Como definiría su expectoración**

1. Expectoro con facilidad un moco fluido

2. Expectoro un moco muy pegajoso y difícil de eliminar ______
