# Supplementary material for: Association of the CFTR gene with asthma and airway mucus hypersecretion
Source: PLoS One. 2021 Jun 4;16(6):e0251881. doi: 10.1371/journal.pone.0251881 (PMC8177500; doi:10.1371/journal.pone.0251881)
Supplement: S2 Appendix — (ZIP) [file pone.0251881.s002.zip › en_statement sample representativeness.pdf]

Date: 03/15/2021

Manuscript Number: PONE-D-20-37886

Title of Article: **Association of the *CFTR* gene with asthma and airway mucus hypersecretion**

Name of the Corresponding Author: **Astrid Crespo-Lessmann**

STATEMENT OF STUDY SAMPLE SIZE

Chronic bronchial mucosal hypersecretion, as a distinct clinical entity, may lead to increased morbidity and mortality from asthma. Insufficiently characterized in asthma studies, this hypersecretion has been correlated with greater severity, deteriorated lung function, a greater number of exacerbations, and a poorer response to glucocorticoid anti-inflammatory treatment. Various pathophysiological mechanisms have been proposed as the cause of bronchial hypersecretion in patients with asthma, including possible genetic causes. This study hypothesizes that the combination of asthma and being a carrier of genetic variants (mutations or polymorphisms) in the *CFTR* gene would cause a variant or phenotype of asthma: asthma with bronchial mucosal hypersecretion. This phenotype, we suggest, is characterized by more severe disease (in terms of control, quality of life, exacerbations and lung function) and by a different inflammatory phenotype than asthma without bronchial hypersecretion. We set the following objectives: (1) to determine the presence of genetic variants of the *CFTR* gene in patients with asthma with and without airway mucus hypersecretion; and (2) to identify the clinical, inflammatory and functional characteristics of the phenotype of asthma with airway mucus hypersecretion (in correctly selected patients). We found that patients with asthma and with mucus hypersecretion may have a different type of disease mechanism, resulting from an intronic polymorphism in the *CFTR* gene (NM_000492.3: c.1680-870T>A) and may have a poorer clinical outcome, characterized by severe disease and poorer asthma control with a non-allergic inflammatory phenotype.
However, despite being a study that included patients with diagnosed asthma (not patients from a database, as done in previous studies) and patients that had undergone extensive clinical, functional and inflammatory testing, the truth is that 4-5% of the general population are estimated to be carriers of cystic fibrosis. To date, some 1,900 genetic variants have been described for the *CFRT* gene, while only some 20 mutations occur with a frequency greater than 0.1% worldwide (1-2). When sample size was calculated, it was aimed to recruit a total of 100 patients (50 patients for each group), assuming a loss of 10%. This number was calculated using the Granmo V7.10 program, setting the type I error at the usual 5% (α=0.05), for a bilateral approximation and for the minimum difference required for a power of 80% or higher (ß=0.2). However, given the low incidence (not known in fact) of patients with asthma and associated bronchial hypersecretion, it was only possible to recruit 39 patients who met the requirements for asthma with bronchial hypersecretion, and 61 patients with asthma without bronchial hypersecretion, so this study may not be representative of a larger population, so its results may have to be validated with a larger sample size.

Astrid Crespo Lessmann, MD, PhD

Principal investigator and corresponding author
