# Supplementary material for: Association of the CFTR gene with asthma and airway mucus hypersecretion
Source: PLoS One. 2021 Jun 4;16(6):e0251881. doi: 10.1371/journal.pone.0251881 (PMC8177500; doi:10.1371/journal.pone.0251881)
Supplement: S3 Appendix — (ZIP) [file pone.0251881.s003.zip › Table S1..docx]

**Supporting information**

Table S1. Summary of the genetic variants in the *CFTR* (cystic fibrosis transmembrane conductance regulator) gene identified in the patients with asthma and the statistic values obtained for the hypersecretion versus non-hypersecretors groups.

| **Genetic variant** | **Hypersecretors**  **(N=38)** | **Non-hypersecretors (N=59)** | **p** |
| --- | --- | --- | --- |
| **c.1680-870T>A** | **21,05%/78,94%**  **(N=8/30)** | **40,67%/59,32%**  **(N=24/35)** | **0.036** |
| **c.1680-871A>G** | 100%/0%  (N=38/0) | 98.3%/1.69%  (N=58/1) | 0.608 |
| **c.1727G>C [p.(Gly576Ala)]** | 94.73%/5.26%  (N=36/2) | 98.3%/1.69%  (N=58/1) | 0.339 |
| **c.2002C>T [p.(Arg668Cys)]** | 92.10%/7.89%  (N=35/3) | 98.3%/1.69%  (N=58/1) | 0.165 |
| **c.2047_2052delAAAAAAinsAAAAG [p.(Lys684Serfs*38)]** | 97.36%/2.63%  (N=37/1) | 100%/  (N=59) | 0.392 |
| **c.2260G>A [p.(Val754Met)]** | 97.36%/2.63%  (N=37/1) | 100%/  (N=59) | 0.392 |
| **c.2506G>T [p.(Asp836Tyr)]** | 97.36%/2.63%  (N=37/1) | 94.91%/5.08%  (N=56/3) | 0.488 |
| **c.2562T>G (p.=)** | 50%/50%  (N=19/19) | 54.23%/45.76%  (N=32/27) | 0.421 |
| **c.2619+3A>G** | 100%/  (N=38/0) | 98.3%/1.69%  (N=58/1) | 0.608 |
| **c.2619+85_2619+86delAT** | 44.73%/55.26%  (N=17/21) | 54.23%/45.76%  (N=32/27) | 0.24 |
| **c.2619+106T>A** | 86.84%/13.55%  (N=33/5) | 86.44%/13.55%  (N=51/8) | 0.604 |
| **c.2909-71G>C** | 97.36%/2.63%  (N=37/1) | 91.52%/8.47%  (N=54/5) | 0.238 |
| **c.2909-92G>A** | 65.78%/34.21%  (N=25/13) | 71.18%/28.81%  (N=42/17) | 0.366 |
| **c.2991G>C [p.(Leu997Phe)]** | 100%/  (N=38) | 98.3%/1.69%  (N=58/1) | 0.608 |
| **c.3139+42A>T** | 100%/  (N=38) | 98.3%/1.69%  (N=58/1) | 0.608 |
| **c.3140-92T>C** | 97.36%/2.63%  (N=37/1) | 89.83%/10.16%  (N=53/6) | 0.16 |
| **c.3285A>T [p.(Thr1095Thr)]** | 97.36%/2.63%  (N=37/1) | 100%/  (N=59) | 0.392 |
| **c.3367+37G>A** | 97.36%/2.63%  (N=37/1) | 100%/  (N=59) | 0.392 |
| **c.3705T>G [p.(Ser1235Arg)]** | 92.10%/7.89%  (N=35/3) | 96.61%/3.38%  (N=57/2) | 0.299 |
| **c.3808G>A [p.(Asp1270Asn)]** | 100%/  (N=38) | 98.3%/1.69%  (N=58/1) | 0.808 |
| **c.3870A>G** | 97.36%/2.63%  (N=37/1) | 91.52%/8.47%  (N=54/5) | 0.238 |
| **c.3874-200G>A** | 94.74%/5.26%  (N=36/2) | 94.91%/5.08%  (N=56/3) | 0.654 |
| **c.3909C>G [p.(Asn1303Lys)]** | 100%/  (N=38) | 98.3%/1.69%  (N=58/1) | 0.608 |
| **c.4137-139G>A** | 57.89%/42.10%  (N=22/16) | 71.18%/28.81%  (N=42/17) | 0.13 |
| **c.4243-20A>G** | 100%/  (N=38) | 98.3%/1.69%  (N=58/1) | 0.608 |
