# Supplementary material for: Association of the CFTR gene with asthma and airway mucus hypersecretion
Source: PLoS One. 2021 Jun 4;16(6):e0251881. doi: 10.1371/journal.pone.0251881 (PMC8177500; doi:10.1371/journal.pone.0251881)
Supplement: S3 Appendix — (ZIP) [file pone.0251881.s003.zip › Table S2.docx]

**Table S2.** Pathological mutations described in the analysed population with asthma. The 4 patients with cystic fibrosis presented with a pathological mutation in heterozygosis.

| **Patient No.** | **Mutation** | **Exon** |
| --- | --- | --- |
| 1 | c.2047_2052delAAAAAAinsAAAAG [p.(Lys684Serfs*38)] | E14 |
| 2 | c.3909C>G [p.(Asn1303Lys)] | E24 |
| 3 | c.1521_1523delCTT [p.(Phe508del)] | E11 |
| 4 | c.350G>A [p.(Arg117His)]; c.1521_1523delCTT [p.(Phe508del)] | E4; E11 |

Patient #4 presented with 2 heterozygous changes: pathological (c.1521_1523delCTT) and change c.350G>A, the latter classified as missense since an amino acid change is predicted, although it is described in the cystic fibrosis database as a genetic variant with variable clinical consequences.
