# Supplementary material for: Association of the CFTR gene with asthma and airway mucus hypersecretion
Source: PLoS One. 2021 Jun 4;16(6):e0251881. doi: 10.1371/journal.pone.0251881 (PMC8177500; doi:10.1371/journal.pone.0251881)
Supplement: S3 Appendix — (ZIP) [file pone.0251881.s003.zip › Table S3.docx]

**Table S3.** Polymorphisms associated with the *CFTR* gene according to asthma severity.

| **GENOTYPE** | **Intermittent asthma**  **(N=25)** | **Persistent asthma** | | |
| --- | --- | --- | --- | --- |
|  |  | **Mild**  **(N=25)** | **Moderate**  **(N=11)** | **Severe**  **(N=36)** |
| **c.2506G>T [p.(Asp836Tyr)]*** |  |  |  |  |
| Homozygous majority allele (G/G) | 23.70% | 25.80% | 11.80% | 38.70% |
| Minority allele present (G/T & T/T) | 75% | 25% | 0% | 0% |
| **c.3140-92T>C**** |  |  |  |  |
| Homozygous majority allele (T/T) | 23.30% | 25.60% | 12.20% | 38.90% |
| Minority allele present (T/C & C/C) | 57.10% | 28.60% | 0% | 14.30% |

CFTR: cystic fibrosis transmembrane conductance regulator. * p=0.024; ** p=0.049.
