# Supplementary material for: Association of the CFTR gene with asthma and airway mucus hypersecretion
Source: PLoS One. 2021 Jun 4;16(6):e0251881. doi: 10.1371/journal.pone.0251881 (PMC8177500; doi:10.1371/journal.pone.0251881)
Supplement: S3 Appendix — (ZIP) [file pone.0251881.s003.zip › Table S4.docx]

**Table S4.** Demographic, clinical and functional characteristics of asthma with and without airway mucus hypersecretion.

| **Variables** | **Asthma with hypersecretion**  **(N=39)** | **Asthma without hypersecretion**  **(N=59)** | **p** |
| --- | --- | --- | --- |
| **Age (y)** | 57.43 (11.47) | 49.44 (15.4) | **0.004** |
| **Sex (% women)** | 61.5% | 49.15% | 0.159 |
| **Asthma diagnosis (% adults)** | 76.92% | 71.18% | 0.349 |
| **BMI (kg/m2)** | 27.41 (4.46) | 27.24 (4.93) | 0.864 |
| **Severe asthma (%)** | 58.97% | 23.72% | **0.005** |
| **FEV1/FVC (%)** | 64.39 (13.28) | 69.55 (9.61) | **0.041** |
| **FeNO (ppb)** | 32.45 (25.64) | 39.81 (43.27) | 0.291 |
| **Positive bronchodilator test (%)** | 23.68% | 35.08% | 0.483 |
| **Emergency visits, last 12 months** | 2.46 (3.08) | 1.48 (2.24) | 0.074 |
| **Oral glucocorticoid treatments, last 12 months** | 3.6 (3.7) | 0.86 (1.3) | **0.002** |
| **Medium-high oral glucocorticoid doses (%)** | 74.35% | 64.4% | 0.678 |
| **Rhinitis (%)** | 61.53% | 64.44% | 0.360 |
| **Polyposis (%)** | 20.55% | 8.62% | 0.172 |
| **High quality induced sputum (%)** | 61% | 27.3% | 0.100 |
| **Inflammatory phenotype**  **in induced sputum (%)** | paucigranulocytic: 31.25%  neutrophilic: 12.5%  eosinophilic: 56.25%  (N=16) | paucigranulocytic: 26.32%  neutrophilic: 26.32%  eosinophilic: 47.36%  (N=19) | 0.596 |
| **Positive prick test (%)** | 46.15% | 64.4% | 0.216 |
| **Blood IgE (IU/mL)** | 126.4 (197) | 407.59 (627.6) | **0.003** |
| **Absolute eosinophils in peripheral blood (x10E9/L)** | 0.39 (0.32) | 0.35 (0.27) | 0.491 |
| **Blood polymerase chain reaction (mg/L)** | 4.26 (5.57) | 4.20 (6.56) | 0.969 |
| **Blood fibrinogen (g/L)** | 4.05 (1.02) | 3.98 (0.93) | 0.777 |
| **Blood albumin (g/L)** | 42.21 (3.06) | 44.14 (3.12) | **0.008** |
| **Lymphocytes in induced sputum (%)** | 0.71 % (0.52) | 1.05% (0.38) | **0.023** |
| **ACT <20 (%)** | 58.3% | 29.09 % | **0.021** |
| **AQLQ** | 3.55 (2.63) | 2.6 (2.62) | 0.113 |

Values are reported as means (standard deviation) or percentages, as indicated. ACT=Asthma Control Test; AQLQ=Asthma Quality of Life Questionnaire; BMI=Body mass index; FeNO=fractional exhaled nitric oxide; FEV1=forced expiratory volume in the first second; FVC=forced vital capacity; IgE=immunoglobulin E.
