# Supplementary material for: Association of the CFTR gene with asthma and airway mucus hypersecretion
Source: PLoS One. 2021 Jun 4;16(6):e0251881. doi: 10.1371/journal.pone.0251881 (PMC8177500; doi:10.1371/journal.pone.0251881)
Supplement: Attachment — Submitted filename: ed_ed_Point-by-point-response.doc [file pone.0251881.s004.doc]

**Point-by-point response template**

**Date: 03/15/2021**

**Manuscript Number:** PONE-D-20-37886

**Title of Article: Association of the *CFTR* gene with asthma and airway mucus hypersecretion**

**Name of the Corresponding Author: Astrid Crespo-Lessmann**

**Email Address of the Corresponding Author: acrespo@santpau.cat**

Dear Ritesh Agarwal

Academic Editor

PLOS ONE

Our thanks to both you and the reviewers for valuable suggestions and comments, which we feel have greatly improved the manuscript.

Chronic bronchial mucosal hypersecretion, as a distinct clinical entity, may lead to increased morbidity and mortality from asthma. Insufficiently characterized in asthma studies, this hypersecretion has been correlated with greater severity, deteriorated lung function, a greater number of exacerbations, and a poorer response to glucocorticoid anti-inflammatory treatment. Various pathophysiological mechanisms have been proposed as the cause of bronchial hypersecretion in patients with asthma, including possible genetic causes. This study hypothesizes that the combination of asthma and being a carrier of genetic variants (mutations or polymorphisms) in the *CFTR* gene would cause a variant or phenotype of asthma: asthma with bronchial mucosal hypersecretion. This phenotype, we suggest, is characterized by more severe disease (in terms of control, quality of life, exacerbations and lung function) and by a different inflammatory phenotype than asthma without bronchial hypersecretion.

We set the following objectives: (1) to determine the presence of genetic variants of the *CFTR* gene in patients with asthma with and without airway mucus hypersecretion; and (2) to identify the clinical, inflammatory and functional characteristics of the phenotype of asthma with airway mucus hypersecretion (in correctly selected patients). We found that patients with asthma and with mucus hypersecretion may have a different phenotype and disease mechanism, resulting from an intronic polymorphism in the *CFTR* gene (NM_000492.3: c.1680-870T>A) and may have a poorer clinical outcome, characterized by severe disease and poorer asthma control with a non-allergic inflammatory phenotype.

However, despite being a study that included patients with diagnosed asthma (not patients from a database, as in previous studies) and patients that had undergone extensive clinical, functional and inflammatory testing, the fact of finding just a single polymorphism may lead to spurious associations, among other reasons, because the variant may be in linkage disequilibrium with one or more different variants and, therefore, may constitute a characteristic haplotype. Nonetheless, our findings may represent a useful first step in recognizing, as a distinct clinical entity, bronchial hypersecretion in patients with asthma. The presence of an intronic polymorphism in the CFTR gene in our study seems to influence having a different disease type and mechanism with a poorer clinical outcome, characterized by more severe disease and poorer asthma control and associated with a non-allergic inflammatory phenotype.

***Journal Requirements:***

***1)* Please ensure that your manuscript meets PLOS ONE's style requirements, including those for file naming. The PLOS ONE style templates can be found at**

https://journals.plos.org/plosone/s/file?id=wjVg/PLOSOne_formatting_sample_main_body.pdf and

<https://journals.plos.org/plosone/s/file?id=ba62/PLOSOne_formatting_sample_title_authors_affiliations.pdf>

***Answer:*** Done.

**2. Please include additional information regarding the survey or questionnaire used in the study and ensure that you have provided sufficient details that others could replicate the analyses.**

For instance, if you developed a questionnaire as part of this study and it is not under a copyright more restrictive than CC-BY, please include a copy, in both the original language and English, as Supporting Information.

If the questionnaire is published, please provide a citation to the questionnaire and/or original publication associated with the questionnaire.

***Answer:*** Included patients were administered a questionnaire to evaluate symptoms and type of expectoration. Asthmatic hypersecretion was defined as the presence of cough productive of sputum on most days for at least three months in two successive years.

We have included copies of the questionnaire (in both languages) used to assess bronchial mucus hypersecretion in patients in the appendices. The questionnaire has not been copyrighted.

**3. In your Methods section, please provide additional information about the participant recruitment method and the demographic details of your participants.**

Please ensure you have provided sufficient details to replicate the analyses such as:

a) the recruitment date range (month and year),

b) a table of relevant demographic details,

c) a statement as to whether your sample can be considered representative of a larger population, and

d) a description of how participants were recruited.

***Answer:***

a) The recruitment date range has been included in p.5, methods section, first paragraph.
b) Table 4 shows the demographic, clinical and functional characteristics of asthma with and without airway mucus hypersecretion.
c) It is estimated that 4-5% of the general population are carriers of this disease (CF). To date, some 1,900 genetic variants have been described for the *CFRT* gene, while only some 20 mutations occur with a frequency greater than 0.1% worldwide (1-2). When sample size was calculated, it was aimed to recruit a total of 100 patients (50 patients for each group), assuming a loss of 10%. This number was calculated using the Granmo V7.10 program, setting the type I error at the usual 5% (α=0.05), for a bilateral approximation and for the minimum difference required for a power of 80% or higher (ß=0.2). However, given the low incidence (not known in fact) of patients with asthma and associated bronchial hypersecretion, it was only possible to recruit 39 patients who met the requirements for asthma with bronchial hypersecretion, and 61 patients with asthma without bronchial hypersecretion, so this study may not be representative of a larger population. A comment on this point has been included in the text (see the discussion section, p.10, third paragraph). We have also included a signed statement with this comment (see Appendix 2).
d) The patients included in the study, who came from the outpatient clinics of the hospitals participating in the study, were recruited according to the criteria of the participating physicians. A comment on this has been added in the methods section, p.5, first paragraph.

References:

1. Asencio O, Cobo N, Seculi JL, Casal t, Bosque M. Programa de cribaje neonatal para la fibrosis quística en Cataluña. *Investig Clin* 2001; 4 (suppl I): 82-3.
2. [Javier de Gracia](https://pubmed.ncbi.nlm.nih.gov/?term=de+Gracia+J&cauthor_id=12433335) , [Antonio Alvarez](https://pubmed.ncbi.nlm.nih.gov/?term=Alvarez+A&cauthor_id=12433335), [Fernando Mata](https://pubmed.ncbi.nlm.nih.gov/?term=Mata+F&cauthor_id=12433335), [Luisa Guarner](https://pubmed.ncbi.nlm.nih.gov/?term=Guarner+L&cauthor_id=12433335), [Montserrat Vendrell](https://pubmed.ncbi.nlm.nih.gov/?term=Vendrell+M&cauthor_id=12433335), [Silvia Gadtner](https://pubmed.ncbi.nlm.nih.gov/?term=Gadtner+S&cauthor_id=12433335), et al. Cystic Fibrosis in Adults: Study of 111 Patients. *Med Clin* (Barc) 2002 Nov 9;119(16):605-9.

4. **Thank you for stating the following in the Competing Interests section:**

'I have read the journal's policy and the authors of this manuscript have the following competing interests:

AC has received fees in the last three years for talks at meetings sponsored by Chiesi, Esteve Laboratories, GlaxoSmithKline, Novartis, Ferrer, Zambón and Boehringer Ingelheim, has received travel and attendance expenses for conferences from Novartis, Bial, Teva and FAES Farma and has received funds/grants for research projects from several state agencies and non-profit foundations and from AstraZeneca.

EC reports non-financial support from Astrazeneca, personal fees from Boehringer-Ingleheim, personal fees and non-financial support from Chiesi, non-financial support from Novartis, non-financial support from Menarini, non-financial support from ALK, outside the submitted work.

SB, EdR, ER, CM, NM, AP, SP, JG, AP, CC, JS and MB have no conflicts of interest to declare.

VP has received fees in the last three years for talks at meetings sponsored by AstraZeneca, Boehringer-Ingelheim, MSD and Chiesi, has received travel and attendance expenses for conferences from AstraZeneca, Chiesi and Novartis, has acted as a consultant for ALK, AstraZeneca, Boehringer, MSD, MundiPharma and Sanofi, and has received funds/grants for research projects from several state agencies and non-profit foundations and from AstraZeneca, Chiesi and Menarini.'

a. Please confirm that this does not alter your adherence to all PLOS ONE policies on sharing data and materials, by including the following statement: "This does not alter our adherence to  PLOS ONE policies on sharing data and materials.” (as detailed online in our guide for authors [http://journals.plos.org/plosone/s/competing-interests](about:blank)).  If there are restrictions on sharing of data and/or materials, please state these.

Please note that we cannot proceed with consideration of your article until this information has been declared.

***Answer:*** See information included on p.12, conflicts of interests section.

b. Please include your updated Competing Interests statement in your cover letter; we will change the online submission form on your behalf.

***Answer:*** OK, thank you.

Please know it is PLOS ONE policy for corresponding authors to declare, on behalf of all authors, all potential competing interests for the purposes of transparency. PLOS defines a competing interest as anything that interferes with, or could reasonably be perceived as interfering with, the full and objective presentation, peer review, editorial decision-making, or publication of research or non-research articles submitted to one of the journals. Competing interests can be financial or non-financial, professional, or personal. Competing interests can arise in relationship to an organization or another person. Please follow this link to our website for more details on competing interests: [http://journals.plos.org/plosone/s/competing-interests](about:blank)

**5. One of the noted authors is a group: Emerging Asthma Group.**

In addition to naming the author group, please list the individual authors and affiliations within this group in the acknowledgments section of your manuscript.

Please also indicate clearly a lead author for this group along with a contact email address.

***Answer:*** Done as requested, see Acknowledgements (p.13).

**6. Please include captions for your Supporting Information files at the end of your manuscript, and update any in-text citations to match accordingly. Please see our Supporting Information guidelines for more information:** [**http://journals.plos.org/plosone/s/supporting-information**](about:blank)

***Answer:*** Done as requested, see appendices (p.13).

**Additional Editor Comments:

In addition to the reviewer's comments, the authors should also perform workup for allergic bronchopulmonary aspergillosis and sweat chloride test in the study subjects**

***Answer:*** All patients with allergic bronchopulmonary aspergillosis were excluded. The sweat chloride test could not be performed, first because we do not have this equipment for adults, and second, because when carrying out a massive sequencing genetic study, if the patient had had cystic fibrosis, this would have been detected. Two comments have been added to the manuscript on these points (p.5, methods section, first and second paragraphs).

**Reviewers' comments:**

**Reviewer's Responses to Questions**

**Comments to the Author
1. Is the manuscript technically sound, and do the data support the conclusions?**

The manuscript must describe a technically sound piece of scientific research with data that supports the conclusions. Experiments must have been conducted rigorously, with appropriate controls, replication, and sample sizes. The conclusions must be drawn appropriately based on the data presented.

Reviewer #1: No

Reviewer #2: Yes

2. Has the statistical analysis been performed appropriately and rigorously?

Reviewer #1: No

Reviewer #2: Yes

3. Have the authors made all data underlying the findings in their manuscript fully available?

The [PLOS Data policy](http://www.plosone.org/static/policies.action#sharing) requires authors to make all data underlying the findings described in their manuscript fully available without restriction, with rare exception (please refer to the Data Availability Statement in the manuscript PDF file). The data should be provided as part of the manuscript or its supporting information, or deposited to a public repository. For example, in addition to summary statistics, the data points behind means, medians and variance measures should be available. If there are restrictions on publicly sharing data—e.g. participant privacy or use of data from a third party—those must be specified.

Reviewer #1: No

Reviewer #2: Yes

4. Is the manuscript presented in an intelligible fashion and written in standard English?

PLOS ONE does not copyedit accepted manuscripts, so the language in submitted articles must be clear, correct, and unambiguous. Any typographical or grammatical errors should be corrected at revision, so please note any specific errors here.

Reviewer #1: Yes

Reviewer #2: Yes

 5. Review Comments to the Author

Please use the space provided to explain your answers to the questions above. You may also include additional comments for the author, including concerns about dual publication, research ethics, or publication ethics. (Please upload your review as an attachment if it exceeds 20,000 characters)

Reviewer #1: The study investigates the airway mucus hypersecretors and non-hypersecretors with asthma and aims to correlate genetic variants (mutations or polymorphisms) in the CFTR gene using next-generation sequencing (NGS).
Utilizing pre-defined clinical characteristics the authors undertake a comparative cross-sectional multicentre study including 39 hypersecretors and 61 non-hypersecretors asthma patients

Major comments:
Although the authors follow a thorough protocol for Clinical and demographic data, the statistical methods employed by the authors to study/link the genetic variants in the CFTR gene with the airway mucus secretions are inadequate:

1. The authors use a Multiplicom CFTR panel on illumine MiSeq sequencer to sequence the gene. By calling the SNPs (see minor comments), the authors employ a chi-square test to establish a link between the airway mucus hypersecretors and non-hypersecretors. Although this approach is adequate if one wants to analyze 1 single criterion (here SNPs), this test fails to produce reliable results when multiple SNPs are tested.

As the authors mentioned, they got 50 genetic variants in the CFTR gene. When performing multiple tests, we often encounter the distribution of true positive results to be virtually indistinguishable from the false positive results.
Therefore the authors need to undertake statistical analysis where the significance values that are adjusted for multiple testing. This is needed to control foe the FDR as mentioned above.

It is recommended that the authors re-analyze the SNP data using PLINK software. Care should be taken for issues like population stratification and SNP duplications, SNP strand issues, etc.

***Answer:*** We downloaded PLINK to perform the analysis suggested by the reviewer. However, we were unable to use the software because it is a very complex CMD.exe and we have insufficient experience to be able to import, analyse and interpret the results. Therefore, in an attempt to respond to these comments in the manuscript, we have included a paragraph in the manuscript (p.7, statistical analysis section).

Minor Comments:
The authors mention “data interpretation using computerized systems and
databases incorporated in analytical software” on line 201. This statement is highly ambiguous and needs clarification:

1. what is the sequencing depth/coverage?
2. what is panel (horizontal) coverage of the CFTR gene?
3. how were the SNPs called? Mapping to reference? What version of human genome? SNP filtering criteria?

***Answer:*** We agree with the minor comments made by reviewer #1. For this reason, we have included information on p.6, see “Study of genetic variants in the CFTR gene“, first paragraph*.*

Reviewer #2: Line no. 204 – Please mention the method by which zygosity analysis was performed.

**Answer:** We agree with the comments made by reviewer # 2. We have added a sentence with the requested information in the corresponding paragraph.

Line no. 223 - It is written that 50 genetic variants were found in CFTR gene. These were found in how many patients. Also mention if any sample had more than 1 variants and mention its implications in the current study.

***Answer:*** In Table 1, we have included the frequency of these genetic variants in the majority and minority allele of the studied population and have also modified the first paragraph of the genetic results section (p.7)

Line no. 300 - The SNP cant be linked directly to the causation. The samaple size is small to come to this conclusion. The authors can describe it as a plausible association rather than giving it as a definite linkage.

***Answer:*** Corrected in the discussion (p.8, first paragraph) and in the conclusions (p.12, first paragraph)

Line no. 374 - No healthy controls were taken for comparison. So, this statement cant be valid.

***Answer:*** This statement is not correct, it’s true.

We have therefore changed the original sentence: “It is estimated that around 4%-5% of the general population are CF carriers, and the fact that the NGS of 98 samples from patients with asthma resulted in 4% being CF carriers demonstrates the internal validity of our genetic study.”

To the following sentence (and have added a new citation (32) in the references section):

“It is estimated that around 1 in 25-30 Caucasians are carriers of CF mutations – a higher frequency than for other ethnic and racial groups (7,11,32). The fact that the NGS of 97 patients from our sample with asthma confirmed 4% to be CF carriers would tend to corroborate the internal validity of our genetic study” (p.10, last paragraph, discussion section).

Line no. 377 - In the supplementary material excel, it was shown than the mulitple SNPs are present in single patient. How do you explain them and what is there significance.

***Answer:*** The multiple SNPs present in a single patient is explained by the fact that not all the patients in the study were Caucasian. This information has been included (p.11, final paragraph, discussion section).

6. PLOS authors have the option to publish the peer review history of their article ([what does this mean?](https://journals.plos.org/plosone/s/editorial-and-peer-review-process#loc-peer-review-history)). If published, this will include your full peer review and any attached files.


If you choose “no”, your identity will remain anonymous but your review may still be made public.


**Do you want your identity to be public for this peer review?** For information about this choice, including consent withdrawal, please see our [Privacy Policy](https://www.plos.org/privacy-policy).

Reviewer #1: No

Reviewer #2: **Yes:** Shivaprakash M Rudramurthy

[NOTE: If reviewer comments were submitted as an attachment file, they will be attached to this email and accessible via the submission site. Please log into your account, locate the manuscript record, and check for the action link "View Attachments". If this link does not appear, there are no attachment files.]

While revising your submission, please upload your figure files to the Preflight Analysis and Conversion Engine (PACE) digital diagnostic tool, <https://pacev2.apexcovantage.com/>. PACE helps ensure that figures meet PLOS requirements. To use PACE, you must first register as a user. Registration is free. Then, login and navigate to the UPLOAD tab, where you will find detailed instructions on how to use the tool. If you encounter any issues or have any questions when using PACE, please email PLOS at [figures@plos.org](mailto:figures@plos.org). Please note that Supporting Information files do not need this step.
